# Negative outcome in cutaneous *Mycobacterium marinum* infection treated with surgical intervention: Two-case report

**DOI:** 10.1097/MD.0000000000040179

**Published:** 2024-10-18

**Authors:** Bo Sang, Xiujiao Xia, Zehu Liu

**Affiliations:** a Department of Dermatology, Hangzhou Third People’s Hospital, Hangzhou Third Hospital Affiliated to Zhejiang Chinese Medical University, Hangzhou, China.

**Keywords:** case report, *Mycobacterium marinum*, surgical intervention

## Abstract

**Rationale:**

*Mycobacterium marinum* (*M marinum*), a slow-growing nontuberculous mycobacterium (NTM), is widely distributed in aquatic environments. It is a well-known cutaneous pathogen, which causes sporotrichosis-like lesions.

**Patient concerns:**

In this report, we describe 2 cases of subcutaneous *M marinum* infection. Both patients underwent several surgical procedures at local hospitals, and despite optimal surgical site healing, new lesions appeared in adjacent sites.

**Diagnoses:**

Based on NTM culture, identification by gene sequencing, and matrix-assisted laser desorption ionization time-of-flight mass spectrometry, the diagnosis of subcutaneous NTM infection was confirmed.

**Interventions:**

The patients were treated with oral rifampicin 0.45 g/day and clarithromycin 1 g/day and oral doxycycline hydrochloride capsules (200 mg/day), respectively.

**Outcomes:**

Both patients were treated for 8 and 5 weeks, respectively, and the lesions healed.

**Lessons:**

Surgical debridement cannot compete with or impede NTM lymphatic spread; antimicrobial therapy is the first choice for the treatment of *M marinum* infections.

## 1. Introduction

Human cutaneous *Mycobacterium marinum* (*M marinum*) infection is mainly associated with aquatic exposure.^[1]^ The disease usually presents as a solitary papule or nodule, sometimes as a sporotrichoid lymphocutaneous pattern. Due to the lack of diagnostic facilities, primary hospitals often use surgery as an early empirical treatment. Herein, we report 2 cases of cutaneous *M marinum* infection, both of whom underwent several surgical treatments in other hospitals, but their lesions still showed lymphatic migration.

## 2. Case presentation

### 2.1. Case 1

**Figure 1.**
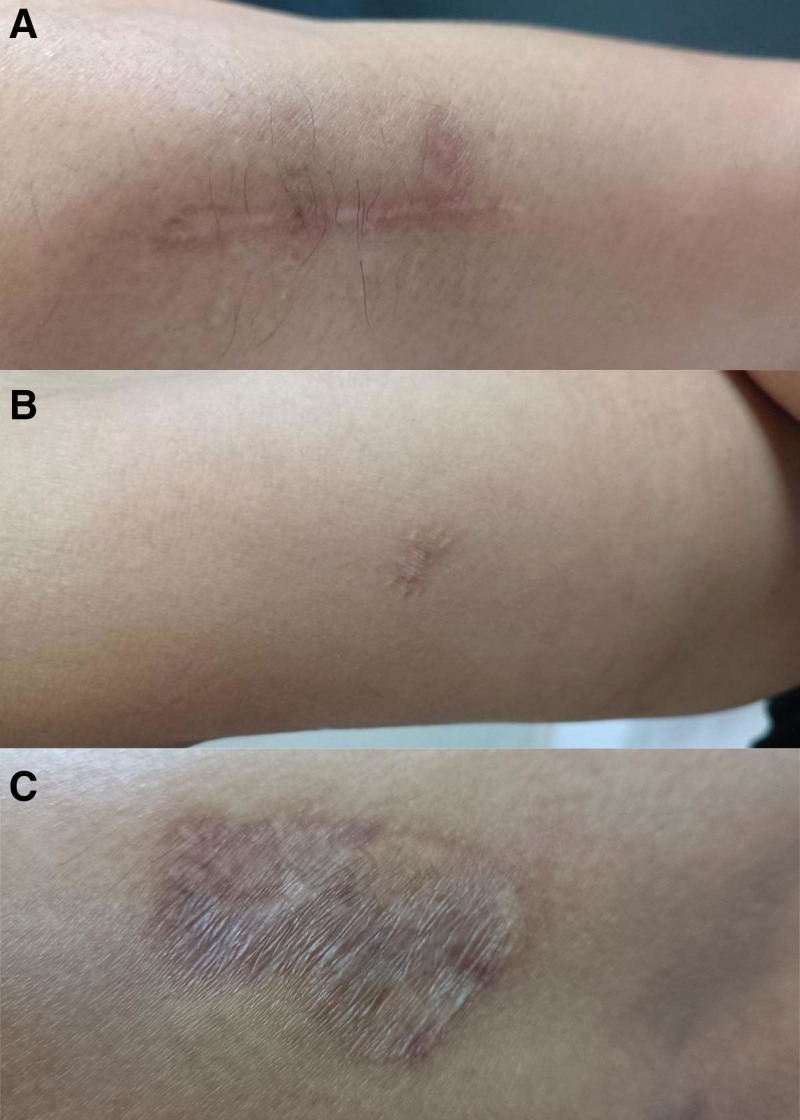
Multiple scars in case 1: (A) right forearm after the first surgery, (B) right back arm after the second surgery, and (C) self-healing of lesion adjacent to the second surgical site.

**Figure 2.**
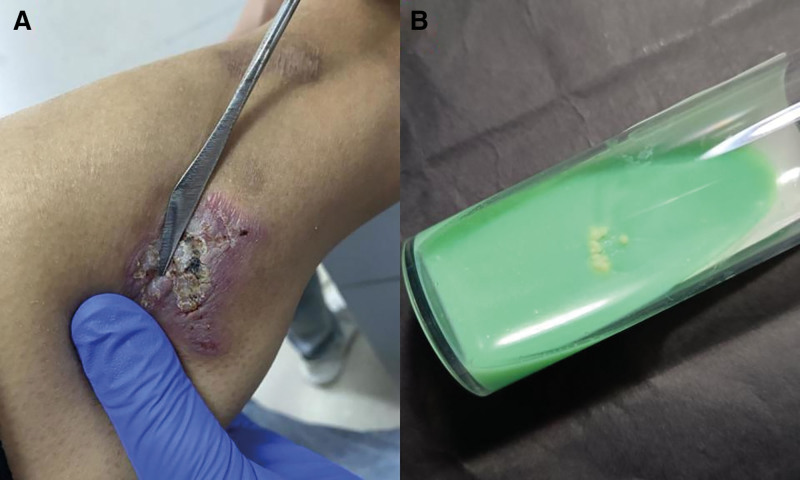
(A) New lesion on the right back arm in case 1. (B) *Mycobacterium marinum* colonies on Lowenstein-Jensen solid medium after 17 days of incubation at 25 °C.

A 46-year-old male seafood trader presented to our outpatient department. He complained of erythematous plaques on his right arm. He had no history of trauma and noted that the lesions began as a papule on the extensor part of his right forearm 8 years previously, with gradual enlargement. The lesion was surgically removed at a local hospital, 1 week after onset. One month after surgery, new lesions appeared on the posterior arm: 2 on the extensor surface and 1 on the flexor surface. A year ago, the patient presented at another hospital. The flexor surface lesion was surgically removed since sarcoidosis was initially suspected. Shortly thereafter, the surgical site and adjacent extensor surface lesion both healed. On physical examination, a dark red plaque with yellow and black crusts was seen on the proximal region of the right arm. Three scars of varying sizes, 2 located proximally on the arm and 1 distally, were noted (Fig. 1A–1C). In addition, the plaque exuded pus upon palpation (Fig. 2A). The purulent specimen was inoculated onto multiple sets of Lowenstein-Jensen solid media and incubated at 25 °C. After 17 days, tiny yellowish colonies were seen (Fig. 2B). The isolate was identified as *M marinum* by the matrix-assisted laser desorption ionization time-of-flight mass spectrometry (Bruker Daltonik MALDI Biotyper) and the 16S ribosomal RNA sequencing (GenBank accession number OR999568). The patient was treated with oral rifampicin 0.45 g/day and clarithromycin 1 g/day for 8 weeks. A 6-month follow-up revealed that the skin lesion had resolved.

### 2.2. Case 2

**Figure 3.**
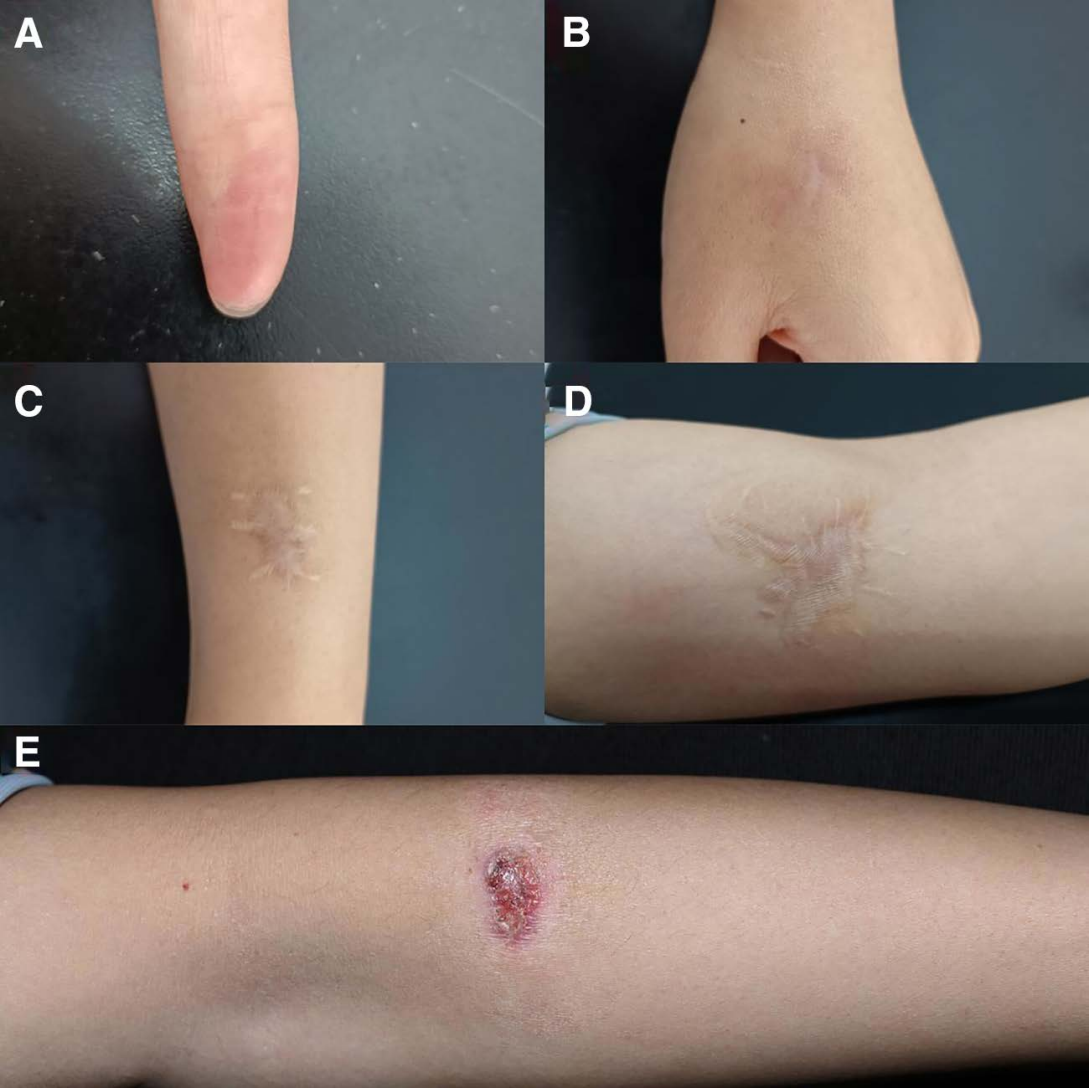
Clinical images of case 2: (A) the left index finger after self-healing, (B–D) the postoperative scars, and (E) new lesion on the left upper extremity.

A 26-year-old female seafood trader presented with a 3-year history of ulcerative plaques on her left arm. The patient recalled that her left index finger had been pricked by a fishbone before the lesions appeared. A red papule appeared on the wound site and evolved into painless erythematous nodules that spread upwards along the dorsum of the hand. That year, there were 4 new skin lesions on the left arm, which were surgically removed at a local hospital due to suspected infectious granuloma. The original lesion on the finger healed, and of the 4 sites removed surgically, only 1 failed to heal. A dermatological examination revealed 3 surgical scars and an ulcerative plaque distributed along the lymphatic vessels on the left forearm (Fig. 3A–3E). Biopsies taken from the plaque were sent for histopathological examination and mycobacterial culture. Histopathology of the dermis revealed chronic infectious granuloma. The fluid and tissue cultured on Lowenstein-Jensen solid medium at 25 °C for 13 days revealed colonies of *Mycobacterium spp*. The isolate was identified as *M marinum* by matrix-assisted laser desorption ionization time-of-flight mass spectrometry and 16S ribosomal RNA sequencing (GenBank accession number PP000802). The patient was treated with oral doxycycline hydrochloride capsules (200 mg/day) for 5 months until the ulcerative plaque resolved.

### 2.3. Ethical approval and consent

Written and informed consent was obtained from this patient. The study was approved by the Medical Ethics Committee at the Department of Dermatology, Hangzhou Third People’s Hospital.

## 3. Discussion

*M marinum* is a known pathogen that can cause skin and soft tissue infections in persons working with contaminated water, such as fish handlers.^[2]^ Single or multiple papules, nodules, or plaques often appear at the injury site within 2 to 8 weeks following inoculation with *M marinum*.^[3]^ About a quarter to a third of cases may develop into a sporotrichoid pattern.^[4]^ *M marinum* grows slowly in culture, so it cannot be detected in conventional, short-term bacterial cultures. Even in outbreak settings, the sensitivity of culture can be as low as 50%.^[5]^ In short, the diagnosis of *M marinum* infection is challenging due to the variable clinical presentation and rarity of *M marinum* infections, especially in primary hospitals without diagnostic facilities.

There are currently no standard treatment protocols for *M marinum* infections. Due to a lack of diagnostic facilities, our 2 patients were initially suspected of having cutaneous sarcoidosis and infectious granuloma, respectively, and were treated with surgical debridement. Unfortunately, their lesions still showed lymphatic migration. These 2 cases show that surgical debridement is locally effective but does not achieve a complete cure. As Zeno paradox shows,^[6]^ Achilles can never catch up with the Tortoise because the Tortoise is always 1 step ahead of the others. This suggests that surgical debridement cannot compete with or impede lymphatic spread, much like the underlying argument of the Zeno paradox. Hence, antimicrobial therapy is the first choice for the treatment of *M marinum* infections. Attention should be paid to the capacity building of nontuberculous mycobacterium infection diagnosis to improve the level of diagnosis in primary hospitals.

## Author contributions

**Data curation:** Bo Sang.

**Investigation:** Bo Sang.

**Methodology:** Bo Sang.

**Writing – original draft:** Bo Sang.

**Conceptualization:** Xiujiao Xia.

**Formal analysis:** Xiujiao Xia.

**Validation:** Xiujiao Xia, Zehu Liu.

**Writing – review & editing:** Xiujiao Xia, Zehu Liu.

**Funding acquisition:** Zehu Liu.
